# Efficacy of intranasal administration of artesunate in experimental cerebral malaria

**DOI:** 10.1186/1475-2875-13-501

**Published:** 2014-12-16

**Authors:** Anne Marijon, Guillaume Bonnot, Anthony Fourier, Coralie Bringer, Adeline Lavoignat, Marie-Claude Gagnieu, Anne-Lise Bienvenu, Stéphane Picot

**Affiliations:** Université Claude Bernard Lyon 1, Malaria Research Unit, SMITH, ICBMS, UMR 5246 CNRS-INSA-CPE-UCBL1, 8 avenue Rockefeller, 69373 Lyon cedex 08, France; Hospices Civils de Lyon, Institut de Parasitologie et de Mycologie Médicale (IP2M), Hôpital de la Croix-Rousse, 103 grande rue de la Croix-Rousse, 69317 Lyon cedex 04, France; Hospices Civils de Lyon, Laboratoire de Pharmacologie spécialisée, Hôpital Edouard Herriot, 5 place d’Arsonval, 69437 Lyon cedex 03, France

**Keywords:** Cerebral malaria, Artesunate, Intranasal administration, Pharmacokinetic, Toxicity

## Abstract

**Background:**

Improving management of patients suffering from cerebral malaria is needed to reduce the devastating mortality and morbidity of the disease in endemic areas. Intravenous artesunate is currently the first-line treatment, but the lack of material and skills in the field make it difficult to implement in endemic areas. Intranasal route provides a very easy and direct gateway to blood and brain to deliver medications, by-passing the brain blood barrier. Therefore, it could be helpful and suitable to administer artesunate in the context of cerebral malaria, especially in young children. In this study, intranasal administration of artesunate to rescue from cerebral malaria using a murine model was tested.

**Methods:**

CBA/J mice infected with *Plasmodium berghei* ANKA strain received artesunate (20 mg/kg) or a placebo solution intranasally, either on day 5, 6 or 7 post-infection, during a controlled, blinded, randomized trial. Primary endpoint was mortality on day 12 post-infection. Secondary endpoints were parasitaemia and clinical stage. Pharmacokinetics data following administration were collected in blood and brains of treated mice. Local toxicity was evaluated by histopathologic examination of brain and nasal sections in blinded manner.

**Results:**

Intranasal administration of artesunate dramatically reduced the mortality rate (p < 0.001), preventing death in most cases. Parasitaemia loads decreased by 88.7% (61.8-100%) within 24 hours after administration. Symptoms of cerebral malaria were prevented or reversed. Dihydroartemisinin was detected in mice blood and brain within 15 minutes of intranasal administration. No direct nasal or brain toxicity was detected.

**Conclusion:**

Intranasal delivery is an efficient route to timely and efficiently administer artesunate and therefore may contribute to decreasing malaria-related mortality.

## Background

Malaria still causes an unacceptable mortality rate in endemic areas and is responsible for more than half a million deaths each year, mainly in sub-Saharan Africa and in children under five years [1, 2]. Without treatment, cerebral malaria invariably leads to death, mostly within the first 36 hours. Five to 30% of surviving children may be left with neurological disabilities [3, 4]. Eliminating malaria requires preventing mosquito bites, making accurate diagnosis, administering early treatment with good drugs at the right dose, and future mass vaccination. Considerable progress has been made, but malaria elimination still needs universal access to these strategies. Bed nets are insufficiently used in endemic areas, rapid diagnostic tests and effective drugs are poorly available in remote areas and malaria vaccine is far from being available. Cerebral malaria is an emergency that requires early diagnostic and care in order to prevent both fatal outcome and risk of neurological sequelae.

Intravenous artesunate is currently the first-line therapy since its superiority over quinine has been demonstrated in large randomized trials [5, 6]. Artesunate is a water-soluble, semi-synthetic, artemisinin derivative. It displays high and fast antiparasitic properties with a broad specificity of action on the various stages of the parasite. Its outstanding ability to rapidly kill circulating ring-stage parasites, preventing their maturation and sequestration in deep organs, including the brain, explains its high efficiency to rescue severe malaria [7–9].

In endemic areas, most patients do not have access to intense care units with trained medical staff; the intravenous route is difficult to implement in the field and even more so in young children. In this context, intravenous administration of artesunate may be delayed or made in a perilous manner and prognosis of patients is even poorer. Rectal administration of artesunate is an alternative route already available [10, 11]. The World Health Organization (WHO) recommends its use as pre-referral treatment during transportation to an appropriate care centre [12]. Some cultural concerns may limit its use as observed in some endemic areas. The intrarectal route allows a systemic absorption without brain-specific targeting. Moreover, a delayed diffusion with variation among individuals are reported [13]. More effective methods are urgently needed to improve the management of severe malaria patients in remote areas of Africa.

The nasal mucosa’s high degree of vascularization and high permeability enable systemic drug concentration via this route, making intranasal (IN) drug administration attractive compared to oral and parenteral routes [14, 15]. The number of drugs administered via the nasal route has increased. Among these, the most popular are analgesics (morphine), migraine treatment (sumatriptan), cardiovascular drugs (propranolol), hormones (progesterone), anti-inflammatory drugs (indomethacin). Recently, the key role of olfactory bulb (OLF) in the pathophysiology of experimental cerebral malaria was demonstrated, providing the first evidence that malaria-infected erythrocytes use OLF as a gateway to the brain [16]. The demonstration of *Plasmodium berghei*-infected red blood cell sequestration in the brain promotes interest in experimental therapeutic cerebral malaria. *Plasmodium berghei* cerebral malaria rodent model is an experimental tool to study new routes of drug administration.

Blood supply to the nasal cavity is provided by branches of the ophthalmic artery (ethmoidal arteries in the olfactory region), the splenopalatine artery, branches of the facial artery and extensive anastomoses in the Kiesselbach’s plexus. The arterial blood flow irrigates a dense bed of capillaries and large venous sinusoids near the respiratory zone. The venous return involves the splenopalatine, facial and ophthalmic veins which drain via the superior vena cava into the right heart chambers, and explain the absence of the hepatic first-pass effect [15]. The pharmacokinetic properties of drugs administered via IN route are dependent on physicochemical properties of compounds and on IN conditions, including mucociliary clearance, solution’s viscosity and volume administered. Variations in inspiratory flow or rhinitis were shown to have only a minor influence on the efficacy of deposition on the turbinate zone [15].

IN drug administration offers a non-invasive method that circumvents the blood–brain barrier (BBB) and provides rapid drug absorption for acute brain insults without systemic side effects. This pathway is believed to involve two general mechanisms. The first is internalization of the drug into the primary neurons of the olfactory epithelium, either by endocytotic or pinocytotic mechanisms, and intracellular axonal transport to the OLF. The second is an extracellular pathway that allows for rapid absorption of the drug across the olfactory epithelial cells, either by transcellular or paracellular mechanisms, followed by uptake into the central nervous system.

It has been shown that molecules can take intracellular and extracellular pathways alongside olfactory and trigeminal primary neurons located in the nasal mucosa. From these singular neuronal extensions, drugs can directly reach the OLF and the brainstem, followed by uptake into the central nervous system.

IN route allow substances direct access to the brain. Molecules might also pass in systemic circulation since nasal cavity is highly vascular. Subsequently, IN route allows timely and efficiently delivery of anti-malarial drugs during cerebral malaria. While a preliminary study reported years ago that prophylactic IN administration of dihydroartemisinin was as effective as the intraperitoneal route in mice [17], this route has never been tested further.

The aim of this study was to test safety, pharmacokinetic and efficacy of IN administration of artesunate in a murine model of cerebral malaria. IN administration of artesunate improved survival and symptoms of mice suffering from severe malaria. It rapidly led to a decrease of blood parasitaemia. Artesunate and dihydroartemisinin were detected in blood within 15 minutes of administration and no significant local toxicity was observed. These results support the evidence for a high efficiency of artesunate delivered via the IN route.

## Methods

### Animal model

Animal experiments were conducted in agreement with ethical and general rules for animal protection. The experimental protocol was approved by the Committee of Ethics of animal experiments of Lyon 1 University, France (BH2012-82v2). Severe malaria was induced in female CBA/J mice, weighing 18–20 g (Janvier Labs, Saint Berthevin, France) by intraperitoneal inoculation of 10^6^*P. berghei ANKA* parasitized erythrocytes stored into frozen nitrogen. Despite some differences, this model is the most widely used animal model to study human cerebral malaria since it shares some neurological signs and histopathological features with it [18, 19]. All mice had free access to standard laboratory non-sterile food and tap water *ad libitum*.

### Efficiency study

A blinded, randomized trial *versus* placebo was conducted. On day 5 post-infection (pi), 54 mice were randomized into four groups. A randomization table was edited by an external experimenter who provided the solution (artesunate or placebo) to administer intranasally to each mouse and then did not participate in other steps of the study. Three groups received a single IN administration of artesunate (Guilin Pharmaceutical, Guangxi, China, 20 mg/kg) either on day 5 pi, 6 pi or 7 pi, and one control group received a placebo solution intranasally either on day 5 pi, 6 pi or 7 pi. Artesunate was prepared following supplier instructions for intravenous use with sodium bicarbonate and saline solution. Placebo was made up in the same way without artesunate powder. IN deliveries (6 μl in each nostril) were performed by pipetting after complete anaesthesia with xylazine (3.75 mg/kg) and ketamine (75 mg/kg). Mice were maintained in standing position until they recovered normal ventilation. Primary endpoint was survival rates at day 12 pi. Secondary endpoints were parasitaemia courses and clinical evaluations.

Determinations of parasitaemia were done onto thin blood smears made by vein tail incisions and stained by Giemsa 10%. Parasitaemias were calculated as the arithmetic mean of two independent readings by trained operators (by counting the number of red blood cells (RBCs) parasitized by viable parasite among at least 10,000 RBCs). Discrepancies were resolved by a third reading and consensus between readers.

Bienvenu clinical score was used for clinical monitoring [20]. Stage 0 indicated asymptomatic mice; stage 1, unspecific symptoms (ruffled fur); stage 2, ruffled fur and motor impairments; stage 3, hemiplegia and/or respiratory distress; and, stage 4, coma and/or convulsions.

All mice were euthanized on day 12 pi according to the protocol defined by the Committee of Ethics. The randomization table was disclosed after the collection of all data. Mice with parasitaemia lower than 0.01% (approximately 10^3^ parasites per μl) on day 5 pi were discarded from the analysis. Results shown here are consistent with those from three previous experiments.

### Pharmacokinetic study

Forty-five mice were randomly divided into 15 experimental groups of three animals each. Ten groups were parasitized (as previously described) and five groups were kept healthy in order to assess the impact of the disease on drug properties. Mice received artesunate (Guilin Pharmaceutical, Guangxi, China, 20 mg/kg) intranasally on day 5 pi and were sacrificed at predetermined time-point by cervical dislocation (15 min, 30 min, 45 min, 1 hr, 2 hr, 3 hr, 4 hr, 6 hr, 8 hr, and 16 hr post-dosing). After euthanasia, blood samples and brains were immediately collected. Blood samples were collected into heparinized tubes (Vacutainer, Becton Dickinson Diagnostics, Franklin Lakes, USA) and immediately centrifuged to separate plasma. Plasma and brains were kept frozen at −80°C until drug quantification. Drug extraction was done by mixing plasma or brain homogenates with acetonitrile organic solvent. After centrifugation, supernatants were collected and analysed. Artesunate and its active metabolite, dihydroartemisinin, concentrations were determined by ultra performance liquid chromatography with mass tandem detector (UPLC-MS/MS) method on a Cortecs UPLC column C18 (1.6 μm-2.1 × 75 mm, Waters, Milford, USA). The run time of each sample was 4 min. Detection was based on multiple reaction monitoring with precursor-to-product ion transitions m/z 402.37-267.17 (artesunate), m/z 302.30-163.15 (dihydroartemisinin) and m/z 320.26-247.08 (chloroquine, used as internal standard).

### Toxicity study

Local toxicity after IN administration of artesunate was assessed by histopathology performed in blind manner in collaboration with ANIPATH laboratory (Lyon 1 University, France). Nine healthy CBA/J mice were treated intranasally (as previously described) by either a placebo solution or a solution of artesunate (Guilin Pharmaceutical, Guangxi, China, 20 mg/kg). Mice were sacrificed either 30 min (three control mice and three treated mice) or two days (three treated mice) post-dosing. Tissue samples (brain and face) were immediately collected and post-fixed into formaldehyde 4%. Face samples were decalcified in EDTA solution (10%) for 14 days. After embedment in paraffin and cutting, slides were stained by haematoxylin phloxine saffron (HPS). Sections were examined by a trained veterinary pathologist in blind manner of the group of each mouse.

### Statistical analysis

Statistical analyses were performed using R software version 3.0.2. All values are expressed in means (+/− SEMs). Cumulative survival rates were calculated according to Kaplan-Meier method and groups were compared using the log-rank test. Parasitaemia courses were compared by analysis of variance ANOVA or non-parametric Kruskal-Wallis test when variances were unequal. Parasitaemia before and 24 hours after treatment were compared by paired student *t*-test. p-value <0.05 was considered to be statistically significant.

## Results

### Intranasal administration of artesunate kills the parasite and saves the mice

**Figure 1:**
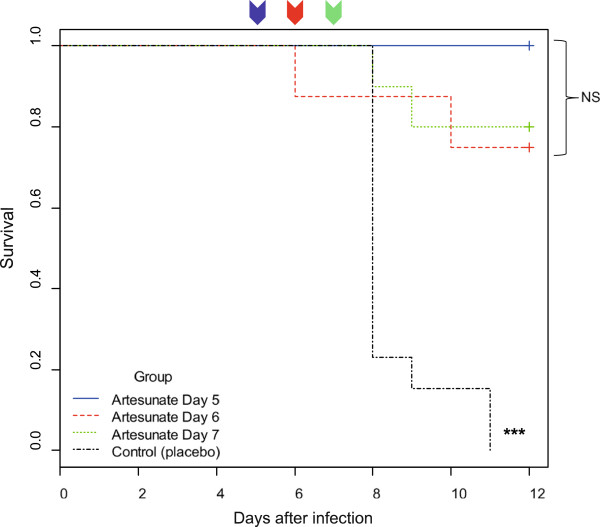
**Cumulative survival analysis (Kaplan-Meier method).** On day 5 pi, 6 pi or 7 pi, mice received a single IN administration of artesunate (20 mg/kg). A control group received IN placebo on day 5 pi, 6 pi or 7 pi. The endpoint was day 12 pi. The log-rank test revealed a significant reduction of the mortality in all treated groups compared with control group (day 5 pi: p = 1.58 10^−7^, day 6 pi: p = 8.89 10^−4^, day 7 pi: p = 4.92 10^−5^). The efficiency of the treatment was similar in the three treated groups (p > 0.05). ***: p <0.001, NS: non-significant, coloured arrows indicates the time of treatment for each group, black line: control group, blue line: mice treated at day 5, red line: mice treated at day 6, green line: mice treated at day 7.

CBA/J mice infected with *P. berghei* ANKA provide a highly reproducible disease leading to death on days 7–8 pi. The trial was blinded and randomized. Mice were treated by one single administration of IN artesunate or placebo either on day 5 pi, day 6 pi or day 7 pi. Primary endpoint was survival rate on day 12 pi. As expected, death of control mice mostly occurred on day 8 pi. Treatment with IN artesunate resulted in a highly significant improvement in survival (Figure 1) compared with control mice. Most treated mice (26/30) were still alive on day 12 pi. This survival improvement was significant whatever the delay between infection and treatment (log-rank test, treatment on day 5 pi (n = 12): p = 1.58 10^−7^, day 6 pi (n = 8): p = 8.89 10^−4^, day 7 pi (n = 10) : p = 4.92 10^−5^). The efficiency of IN administration of artesunate was similar in all treated groups (p >0.05).

**Figure 2:**
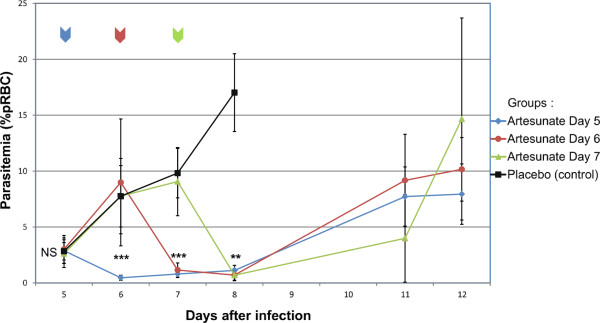
**Parasitaemia courses after intranasal administration of artesunate.** On day 5 pi, 6 pi or 7 pi, mice received a single IN administration of artesunate (20 mg/kg). A control group received IN placebo solution either on day 5 pi, 6 pi or 7 pi. In all treated groups, the treatment administration led to a significant decrease of parasitaemia by 88.7% (61.8-100%) within 24 hours. Parasitaemia were compared using ANOVA on day 5 pi and Kruskal-Wallis test on day 6 pi, 7 pi and 8 pi. In control group, mice were mostly dead on day 8 pi (10/13) and none of them was alive on day 11 pi. ***p <0.001, **p <0.01, NS: non-significant coloured arrows indicates the time of treatment for each group, black line: control group, blue line: mice treated at day 5, red line: mice treated at day 6, green line: mice treated at day 7.

Parasitaemia courses for each group are shown in Figure 2. Before treatment (on day 5 pi), mean parasitaemia was 2.8 +/− 1.0% and parasitaemia levels were similar in all groups (ANOVA, p = 0.893). Parasitaemia of mice treated on day 5 pi decreased by 84.2% (61-100%) within 24 hours of a single artesunate IN administration (mean parasitaemia on day 6 pi = 0.46 +/− 0.23%. On day 6 pi, mice that had not yet been treated (from groups treated on day 6 pi, 7 pi or placebo) showed mean parasitaemia of 8.1% +/−3.8%. Parasitaemia of mice treated on day 6 decreased by 86.8% (22.6-100%) within 24 hours of a single artesunate IN administration (mean parasitaemia on day 7 pi = 1.15 +/− 0.64%).

On day 7 pi, mice that had not yet been treated underwent mean parasitaemia of 9.5 +/−2.6%. Parasitaemia of mice treated on day 7 pi decreased by 92.1% (66.7-100%) within 24 hours of a single artesunate IN administration (mean parasitaemia on day 8 pi = 0.69 +/− 0.49%).

**Figure 3:**
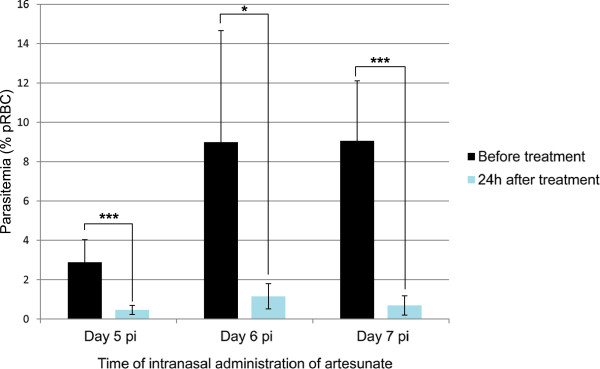
**Parasite clearance 24 hours after intranasal administration of artesunate.** On day 5 pi, 6 pi or 7 pi, mice received a single IN administration of artesunate (20 mg/kg). Parasitaemia before and 24 hours after treatment were compared using paired t student test. Parasitaemia decreased by 84.2% in mice treated on day 5 pi (p = 5.6 × 10^−6^), by 86.8% in mice treated on day 6 pi (p = 0.016) and by 92.1% in mice treated on day 7 pi (p = 3.1 × 10^−5^). *: p <0.05, ***: p <0.001.

Overall, a single IN administration of artesunate in parasitized mice resulted in a decrease of 88.7% (61.8%-100%) of parasitaemia loads within 24 hours. As suspected, parasites escaped rapidly the single dose of artesunate and parasitaemia lastly increased progressively until day 12 pi (Figure 3). The slope of parasite growth was lower than before treatment, and delayed mortality by five to seven days.

### Clinical presentation and outcome of infected mice

**Figure 4:**
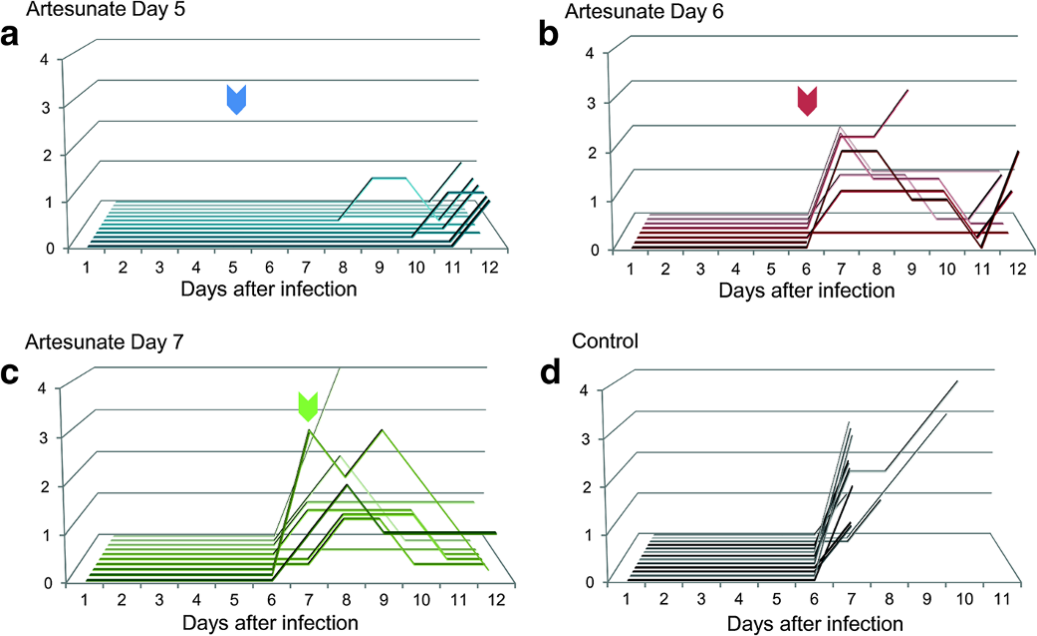
**Clinical monitoring after intranasal administration of artesunate.** On day 5 pi **(a)**, 6 pi **(b)** or 7 pi **(c)**, mice received a single IN administration of artesunate (20 mg/kg). A control group received IN placebo solution **(d)** either on day 5 pi, 6 pi or 7 pi. Compared to control group, IN administration of artesunate led to the absence of symptoms development (when treatment had been administered on day 5 pi) or to a reversion of symptoms (when treatment had been administered on day 6 pi or 7 pi). Bienvenu clinical staging scale: Stage 0: asymptomatic mice; stage 1: unspecific symptoms (ruffled fur); stage 2: ruffled fur and motor impairments; stage 3: hemiplegia and/or respiratory distress; stage 4: coma and/or convulsions. Coloured arrows indicates the time of treatment for each group. Each line represents one mouse.

All mice exhibited a clinical stage 0 (indiscernible symptoms) on day 5 pi. Seventy-seven per cent of the placebo group mice (control) died on day 8 pi (10/13) and 100% were dead at the endpoint. Mice treated once at day 5 pi did not develop severe symptoms until the end of the experiment and all showed a favorable outcome (Figure 4). When mice were treated once on day 6 pi, the trend was to develop moderate symptoms on day 7 pi (clinical stages 1 or 2) and then to recover. One mouse died from acute respiratory distress during anesthesia on day 6 pi and before the administration of the treatment. Most of the mice treated once on day 7 pi exhibited moderate to severe symptoms and progressively recovered after treatment.

### Artesunate is transformed to dihydroartemisin and diffused in blood and brain after IN administration

**Figure 5:**
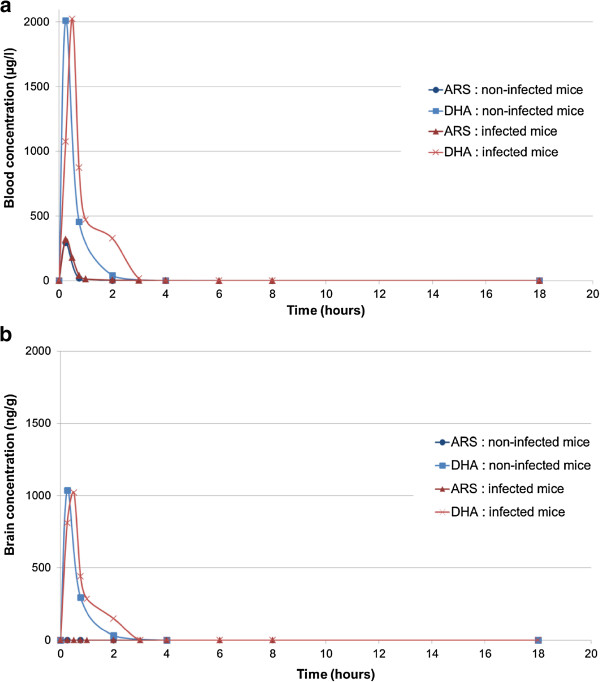
**Artesunate and dihydroartemisinin blood and brain concentrations following intranasal administration of artesunate. (a)** After IN administration, artesunate (ARS) blood concentration reached a very early Cmax (Tmax less than 15 min) and then rapidly converted, showing a short T_1/2_ (close to 15 min). DHA, its active metabolite, reached an early maximum between 15 and 30 min and then decreased with a T_1/2_ close to 15 min. **(b)** No ARS was detected in brains suggesting a prior metabolism before brain diffusion. DHA was detected in brain 15 min after IN administration. DHA brain concentrations globally followed blood concentrations and were about 60% of these.

Mean blood and brain concentration curves of artesunate and its active metabolite, dihydroartemisinin (DHA), in parasitized and uninfected CBA/J mice after IN treatment, are shown in Figure 5. IN artesunate led to high initial blood concentrations; 15 min after administration, mean artesunate blood concentration was 306 μg/l (+/− 83 μg/l). Subsequently, artesunate blood concentration rapidly decreased, with an estimated half-life close to 15 min. There was no difference between uninfected and infected mice. Artesunate was rapidly converted to DHA, which reached an average of 2,018 μg/l (+/− 738 μg/l) blood peak concentration (Cmax) and then rapidly decreased, with an estimated half-life of 15 min. In comparison with healthy mice, there is a trend toward delayed metabolism of artesunate in parasitized mice since DHA curve in parasitized mice seems to be slightly shifted. This subtle trend cannot be definitively interpreted due to the low number of mice per group.

IN artesunate led to DHA brain diffusion. No artesunate was detected in brains of mice treated intranasally with artesunate. This finding suggests initial metabolism of artesunate outside the brain, probably in nasal mucosa and blood where esterase enzymes are present. The average concentration of DHA in brains was about 60% of blood concentrations. It reached a maximum of 1,019 ng/g (+/− 345 ng/g) and followed blood concentration evolution.

### Olfactory tissues and brain are not affected by IN administration of artesunate

Healthy CBA/J mice were treated intranasally by either artesunate (20 mg/kg) or a placebo solution. No relevant clinical signs of local toxicity, as haemorrhage or necrosis, were observed during the experiment. Likewise, none of the mice exhibited general signs of toxicity. No abnormalities in eating, breathing, movement, and behaviour were observed after recovery from anaesthesia.

**Figure 6:**
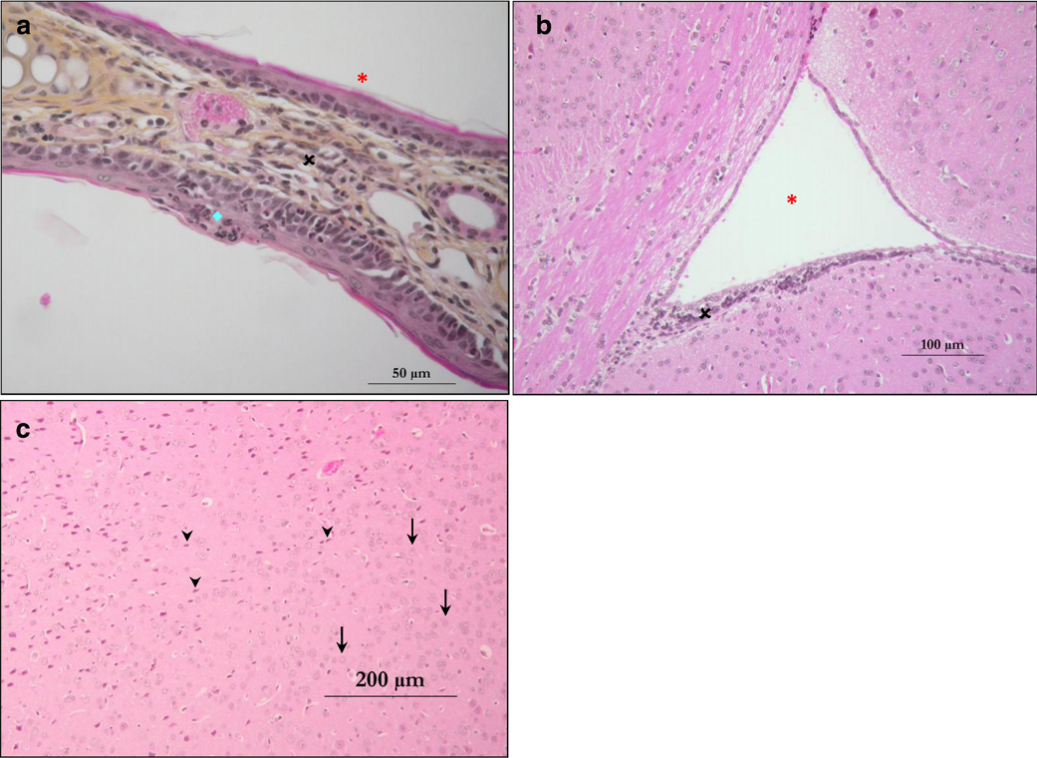
**Haematoxylin phloxine saffron- stained histopathological sections from mice treated intranasally by artesunate (20 mg/kg). (a)** Nasal cavity section. This section shows a nasal fold lined by mucosal epithelium. On the first side, the nasal epithelium is normal with regular ciliary lining cells (red star). On the other side, a little and confined cluster of lymphocytes and neutrophils (blue square) is present and nearby, the epithelium is slightly inflammatory. The underlying *lamina propria* contains a minor lymphoid infiltrate (black cross). **(b)** Brain section through a cerebral ventricule (red star). A lymphoid infiltrate is located in periventricular zone (black cross) without any injury of the neighbouring nervous tissue and the ependimocyte border. **(c)** Brain section. Normal neuron bodies (arrow) are surrounded by neuropil. Dark neurons (arrowhead) are common artifacts characterized as monomorphic contracted and intensively stained neurons. They do not represent degenerating cells.

HPS-stained histopathological sections of brains and nasopharynx were done to assess safety of the treatment at tissue level. IN administration of artesunate did not caused any damage in the respiratory epithelia and olfactory tissue of treated mice, compared to placebo-treated mice. No signs of acute mucosal toxicity [21], such as necrosis or ulceration, was detected. Slight lymphoid infiltrates were observed in the underlying *lamina propria* of the nasal cavity mucosa of four mice (Figure 6a), but this pattern was not correlated with the administration of artesunate (i.e., it was observed in both treated and placebo mice).

Histopathological sections of brains revealed no significant signs of acute neurotoxicity. Some periventricular lymphoid limited infiltrates, were seen in all brains with no correlation with the type of treatment received by the mouse (Figure 6b). One mouse, euthanized 30 min after IN artesunate, had multifocal gliosis (proliferation of astrocytes). This observation is not consistent with a possible toxicity of IN artesunate. Gliosis is a scar of brain parenchyma injury which may appear two to ten days after cerebral tissue damage [22]. In all brains, many artifacts known as dark neurons contracted and intensely stained neurons were observed (Figure 6c). This common artifact may be due to *post-mortem* manipulation of brain tissues prior to fixation and cannot be interpreted as dying cells [23–25]. Moreover, since it was observed in all mice, it is unlikely to be related to the IN administration of artesunate. In summary, no significant toxicity was observed in brains and nasal tissues of mice treated intranasally with artesunate, compared with placebo-treated mice.

## Discussion

In cases of severe malaria, children may die within the first 24–36 hours of the disease [6]. This short period of time is one of the obstacles to a significant reduction in malaria mortality. A pre-referral treatment suitable for tropical and remote conditions is clearly needed, and this route has been opened years ago with the artesunate suppositories development, with still limited uses. Children recovering from cerebral malaria are suffering neurological sequelae with considerable medical, social and economic impact. Parasites sequestered in the microcirculation of the brain are responsible for brain injury. The IN route is the shortest and most convenient way to deliver efficient drug to the brain at the earliest time. The major involvement of olfactory bulb in experimental cerebral malaria and its initial injury at the early phases of the disease provides more evidence for using anti-malarial drugs via the IN route [16].

Artesunate has been proven to be the most effective drug to rescue from cerebral malaria in both adults and children [5, 6, 26]. WHO recommends its intravenous administration immediately when symptoms of severe malaria are detected. Intravenous route ensures a reliable bioavailability but it can be the origin of a delayed administration. Parenteral delivery requires skills that are unavailable or not easily reachable in local health centers. In remote endemic areas, the delay before taking a seriously ill and febrile child to a health care facility may often be more than a day [27]. Alternative routes to provide artesunate are available. Oral medication is inconvenient in situations of severe malaria since patients might exhibit loss of consciousness or vomiting. WHO calls for the use of suppositories of artesunate as pre-referral treatment during transport to an appropriate care center [10–12]. Intrarectal route allows a systemic absorption, without brain-specific targeting. Its pharmakokinetic properties are similar to those obtained from oral administration, but time delayed to Cmax, more than one hour, has been reported. A lower bioavailability with high inter-individual variability has been pointed out, compared to intravenous route [13]. Moreover, this method of administration may confront low acceptability in populations of different cultures. IN delivery is an emerging route to deliver drugs, most notably when the brain is specifically targeted [28–31]. In addition, to allow a systemic entry gate, it offers a direct front door to the central nervous system via the olfactory and trigeminal nerve terminations located in the nasal mucosa. It has been shown that molecules can take intracellular and extracellular pathways alongside these singular neuronal extensions to directly reach the brain [32]. Therefore, drugs can sidestep the BBB. It is an easy, non-invasive route requiring no specific skills or equipment, and therefore, could be appropriate in a rescue context for cerebral malaria in the field.

In the present study, the efficiency of IN administration of artesunate in a murine experimental model of severe malaria was assessed. IN route in mouse models raises some issues. Given the small size of the animals and difficulty of handling, IN administration was done by pipetting 6 μl in each nostril under anaesthesia. Therefore, it cannot be excluded that a small proportion of the dose was swallowed or inhaled. However, this may similarly occur during human administration. Anatomical differences between mice and human should be considered [32, 21]. Rodents are obligate nasal breathers and macrosmatic. The relatively higher surface of their nasal cavity and the higher percentage of olfactive epithelium could improve the passage of drugs through their nasal mucosa. The efficiency and the modalities of IN administration need to be precisely assessed in humans.

IN artesunate was highly effective in saving mice, in both early and late stages of the disease. Even administered later than 24 hours before the death of control mice and/or in mice displaying advanced stages of the disease, IN artesunate prevents death in most cases. Parasitaemia levels cuts around 89% within the first 24 hours after a single IN administration of artesunate. This result correlates with decreases close to 70% described in the literature after intraperitoneal administration of artesunate at higher dosages (32 mg/kg or 64 mg/kg) [18]. Thus, IN route proved its efficiency to deliver artesunate. These results are in accordance with Touitou *et al.*[17] who showed that IN administration of DHA when administered in prophylaxis was at least as effective as the intraperitoneal route to save parasitized mice from death.

Pharmacokinetics data showed early diffusion of DHA following IN administration of artesunate. Cmax in blood and brains was obtained 15 to 30 min after treatment. This observation supports the hypothesis of better efficiency of the IN route compared to rectal route. Direct nose-to-brain drug passage may have occurred when artesunate is administered intranasally but high levels of DHA were found in treated mice brains. Jain *et al*. [33] investigated IN administration of arthemether-loaded nanolipid carriers on healthy rats, showing a direct nose-to-brain transport, with higher brain to blood ratios compared to intravenous administration.

Artesunate is safe and no serious acute side effects were reported in human trials [5, 6]. Nasal and cerebral toxicity induced by IN artesunate were not detected. Long-term exposure to high doses of artesunate can induce cell toxicity [34]. IN administration allows rapid absorption with a short mucosal exposure to the drug. The early elimination of the drug in blood and brain is similar to blood clearance described for intravenous route.

IN administration of artesunate might be helpful in rapid rescue from death but a recrudescence of parasite always occurs since artemisinin derivatives given alone provide only an approximate 20% cure rate [35]. Rescuing therapy based on an artemisinin derivative should be followed by artemisinin-based combination therapy (ACT). IN artesunate could be used as a pre-referral treatment. The IN administration of ACT deserves to be addressed. Altogether, these data open new opportunities for malaria-rescuing treatment in endemic areas. It is expected that phase I-II clinical trials could be conducted in humans in the near future to confirm that evidence. Devices to administer correctly artesunate are available from different providers in the market, and stability tests will be soon available. Advices for correct drug administration in the patient nostrils will be needed during the design of future clinical trials.

For the first time, it was demonstrated that IN delivery is highly effective to treat experimental murine severe malaria in both early and advanced stages of the disease. This finding provides a hopeful way to improve severe malaria rescue treatment in endemic areas. Indeed, early IN administration of the drug might very easily be done without specific skills, to save time over disease progression and reduce malaria-related mortality.

## Abbreviations

ACT: Artemisinin combined therapy
IN: Intranasal
OLF: Olfactory
BBB: Blood–brain barrier
HPS: Haematoxylin phloxine saffron
DHA: Dihydroartemisinin.
